# Supplementary material for: MetaQTL: a package of new computational methods for the meta-analysis of QTL mapping experiments
Source: BMC Bioinformatics. 2007 Feb 8;8:49. doi: 10.1186/1471-2105-8-49 (PMC1808479; doi:10.1186/1471-2105-8-49)
Supplement: Additional File 7 — MetaQTL Package : jar file and tutorial. This Zip archive contains both the MetaQTL JAR file and the files of the tutorial. [file 1471-2105-8-49-S7.zip › org.inra.metaqtl/doc/org/metaqtl/bio/entity/IBioConstants.html]

IBioConstants


|  |  |  |
| --- | --- | --- |
| **Overview** \| **Package** \| **Class** \| **Use** \| **Tree** \| **Deprecated** \| **Index** \| **Help** | |  |
| **PREV CLASS**   **NEXT CLASS** | **FRAMES**    **NO FRAMES**     **All Classes** |  |
| SUMMARY: NESTED \| FIELD \| CONSTR \| METHOD | DETAIL: FIELD \| CONSTR \| METHOD |  |


---


## org.metaqtl.bio.entity Interface IBioConstants

---

``` public interface IBioConstants ```

Class Description Here

**Author:**
:   Jean-Baptiste Veyrieras

---

| **Field Summary** | |
| --- | --- |
| `static IBioAllele[]` | `EMPTY_BIOALLELE_ARRAY` |
| `static BioEntity[]` | `EMPTY_BIOENTITY_ARRAY` |
| `static IBioLGroup[]` | `EMPTY_BIOLGROUP_ARRAY` |
| `static IBioLocus[]` | `EMPTY_BIOLOCUS_ARRAY` |

**Field Detail**

### EMPTY\_BIOENTITY\_ARRAY

```
static final BioEntity[] EMPTY_BIOENTITY_ARRAY
```

---


### EMPTY\_BIOALLELE\_ARRAY

```
static final IBioAllele[] EMPTY_BIOALLELE_ARRAY
```

---


### EMPTY\_BIOLGROUP\_ARRAY

```
static final IBioLGroup[] EMPTY_BIOLGROUP_ARRAY
```

---


### EMPTY\_BIOLOCUS\_ARRAY

```
static final IBioLocus[] EMPTY_BIOLOCUS_ARRAY
```


---


|  |  |  |
| --- | --- | --- |
| **Overview** \| **Package** \| **Class** \| **Use** \| **Tree** \| **Deprecated** \| **Index** \| **Help** | |  |
| **PREV CLASS**   **NEXT CLASS** | **FRAMES**    **NO FRAMES**     **All Classes** |  |
| SUMMARY: NESTED \| FIELD \| CONSTR \| METHOD | DETAIL: FIELD \| CONSTR \| METHOD |  |


---
